# CircCA12 Promotes Malignant Process via Sponging miR-1184 and Upregulating RAS Family in Bladder Cancer

**DOI:** 10.3389/fgene.2021.663982

**Published:** 2021-06-21

**Authors:** Lijuan Jiang, Yanjun Wang, Huancheng Tang, Xiangdong Li, Chaowen Huang, Zhuowei Liu, Fangjian Zhou, Xiaolan Wang, Yonghong Li

**Affiliations:** ^1^Department of Urology, Sun Yat-sen University Cancer Center, State Key Laboratory of Oncology in South China, Collaborative Innovation Center of Cancer Medicine, Guangzhou, China; ^2^Reproductive Center of Medicine, The First Affiliated Hospital of University of South China, Hengyang, China

**Keywords:** circCA12, miR-1184, RAS, sponge, bladder cancer

## Abstract

Circular RNAs (circRNAs) are a panel of non-coding RNAs that mediate the regulation of gene expression, as well as pathological responses. Nonetheless, the function and expression pattern of circRNAs in urinary bladder cancer (UBC) remain unclear. Herein, we examined the function of circCA12 in UBC development. qRT-PCR results demonstrated remarkable circCA12 upregulation in UBC cell lines, as well as tissues. CCK-8, colony formation, and xenograft assays were employed to determine the effect of circCA12 on UBC. Our data illustrated silencing circCA12 repressed the proliferation along with the colony-formation capability of UBC cells. The migration and metastasis potential of UBC cells were remarkably abated *in vivo*, as well as *in vitro* after transfection with si-cirCA12 or sh-circCA12. Moreover, luciferase reporter and RIP assays indicated that circCA12 binds to miRNA-1184 through sponging miRNA, thereby up-regulating the expression of RAS family genes (*NRAS*, *KRAS*, and *HRAS*). In conclusion, the circCA12/miRNA-1184/RAS family was identified as a regulatory axis in UBC progression.

## Introduction

Urinary bladder cancer (UBC) is responsible for about 549,000 new cases, as well as 200,000 deaths every year. It is the sixth most prevalent cancer in men and is ranked ninth among the main causes of cancer-linked deaths (Bray et al., 2018). Reports indicate that it is the most frequent malignant tumor in the urinary system (Antoni et al., 2017). Tobacco use, along with occupational exposure to carcinogens, are the primary predisposing factors of UBC occurrence (Cumberbatch et al., 2018). UBC is classified into different subtypes as per the extension of tumor invasion, namely NMIBC (non-muscle-invasive bladder cancer) and MIBC (muscle-invasive bladder cancer) (Sanli et al., 2017). Individuals with MIBC account for about 20–30% of the total number of UBC cases (Selth et al., 2017). The 5-year survival rate still remains around 60% despite the development of a wide range of treatment approaches including surgical resection and reconstruction, chemotherapy, and radiation therapy (Racioppi et al., 2012). Mounting research evidence opines that a group of molecular and clinicopathological heterogeneous factors regulate the development of UBC (Inamura, 2018). Identification of these factors can aid in the development of several new approaches for diagnosing, classifying, and treating UBC.

Several investigations have documented that circular RNAs (circRNAs) modulate UBC pathogenesis. CircRNAs constitute non-coding RNAs with a covalently closed loop structure, which exist widely in eukaryotic cells (Sanger et al., 1976). Recently, numerous investigations have documented diverse circRNAs in multiple cancer cell lines and tissues (Zhang et al., 2013; Mirzaei and Hamblin, 2020; Nahand et al., 2020; Zou et al., 2020), where they participate in the onset and progress of numerous forms of cancers, e.g., breast cancer (Sang et al., 2019; Zou et al., 2019b; Liu et al., 2020), Esophageal squamous cell cancer (Fan et al., 2019), gastric cancer (Zhang et al., 2017), ovarian cancer (Shabaninejad et al., 2019; Razavi et al., 2021), thyroid cancer (Borran et al., 2020), and gallbladder cancer (Wang et al., 2019). CircRNAs also have potential in regulating the tumor microenvironment (Zou et al., 2019a; Kristensen et al., 2020). Some investigations have postulated that circRNAs act as competing-endogenous RNAs (ceRNAs) through competing with other RNAs to dock miRNAs, a large class of sncRNAs associated with cancer proliferation, differentiation, metastasis, and carcinogenesis. Consequently, they suppress the function of targeted miRNAs (Romero-Cordoba et al., 2014; Li et al., 2015).

Given the promising role of circRNAs in the development of malignant tumor, we designed this study to determine whether endogenous circular RNA can facilitate progression of UBC. It was speculated that circCA12 might modulate the progress of UBC via influencing RAS oncogene expression in a miRNA-mediated approach. Hence, we conducted *in vitro* along with *in vivo* biological and molecular assays to elucidate the responsible mechanism. We established that circCA12 enhances UBC and regulates the carcinogenesis process through the circCA12/miRNA-1184/RAS axis.

## Materials and Methods

Fresh UBC samples and matched non-malignant tissues which met the ethical standards were collected at Sun Yat-sen University Cancer Centre (SYSUCC). Tissues were immediately kept in RNAlater (Ambion, Austin, Texas) after resection. The Helsinki Declaration was strictly adhered to in this study. In addition, the Ethics Committee of SYSUCC approved the study. All participants signed informed consent before the study begun. Furthermore, animal experiments were approved by the Institutional Animal Care and Use Committee of SYSUCC.

### Cell Culture and Treatment

The svhuc1, 5637, RT-112, and BIU-87 cell lines were supplied by the American Type Culture Collection (ATCC, United States). The cell lines were sub-cultured in our laboratory for less than 6 months and maintained as described by the manufacturer. Before use, DNA fingerprinting was used to verify mycoplasma infection and authenticity.

### Colony Formation Assay and CCK-8 Assay

RT-112 and BIU-87 cell lines were planted onto petri dishes and incubated for 14 days. Next, cellular colonies were fixed with 4% PFA and dyed with crystal violet. Transfected cells were then planted into culture plates and inoculated with 10 μl of CCK-8 reagent, followed by incubation in accordance with the protocols described in the CCK-8 assay kit (Dojindo Japan). The absorbance was determined at 450 nm.

### Transfection and qRT-PCR

RNA was isolated using Trizol (Invitrogen, United States). Afterward, M-MLV RT kit (Invitrogen, United States) was employed to synthesize cDNA as described in the manufacturer’s manual. SYBR Premix Ex Taq (Takara) was employed to conduct qRT-PCR assays in a 10 μl total volume including 2 μl cDNA, 5 μl 2 × Master Mix, 0.5 μl Forward Primer (10 μM), 0.5 μl Reverse Primer (10 μM), and 2 μl ddH2O. The amplification protocol involved heating the mixture at 95°C for 10 min, then at 95°C (10 s), and then at 60°C (60 s) for 40 cycles. Results were harvested in three independent wells.

### Luciferase Reporter Assay

3′UTR of HRAS/KRAS/NRAS and circCA12 sequence harboring wide-type (WT) or mutant-type (Mut) docking sites for miR-1184 were created in the pGL3 luciferase vector (Promega, Madison, WI, United States) to obtain circCA12-WT, circCA12-Mut, HRAS/KRAS/NRAS -3′UTR-WT (WT), and HRAS/KRAS/NRAS-3′UTR-Mut (Mut). The predicted miR-1184 binding sites of circCA12, 3′-UTR of HRAS/KRAS/NRAS, was mutated. miR-1184 with locked-nucleic acid (miR-1184-LNA), as well as miR-1184 mimics were inserted into UBC cells accompanied by the reporter plasmid (pRL-TK vector, Promega) via transfection. The activity of luciferase enzyme was then determined using DLR (dual-luciferase enzyme Reporter) assay system kit (Promega) 48 h post transfection, as described by the manufacturer.

### RNA Immunoprecipitation (RIP)

RT-112 UBC cells were co-transfected with MS2bs-circCA12, MS2bs-miRNA-1184, MS2bs-KRAS, MS2bs-NRAS, and MS2bs-HRAS. The Magna RIP RNA-Binding Protein Immunoprecipitation Kit (Millipore) was employed to perform RIP and miR-1184 quantification was done after purification. Anti-Ago2 antibody (Cat No, Millipore) was employed to conduct the RIP assay for Ago2 and the abundance of circCA12, miRNA-1184, KRAS, NRAS, and HRAS was determined after purification.

### Immunohistochemical Analysis (IHC)

Bladder tissues were sectioned into 4-μm-thick slices from embedded blocks. The slices were deparaffinized, rehydrated, and treated with a 90% methanol/3% Hydrogen peroxide solution for 15 min at 37°C. Next, the sections were inoculated in sodium citrate buffer (10 mM sodium citrate; 0.05% Tween 20, pH 6.0) for 5 min at 96°C. Afterward, the sections were blocked with BSA, followed by overnight inoculation with anti-circCA12 antibody (Santa Cruz Biotechnology, Europe; 1:100) at 4°C. Subsequently, they were inoculated at 37°C with a biotinylated secondary antibody for 10 min, then finally with Horseradish Peroxidase (HRP)-streptavidin for 10 min. Results were identified as positive or negative after DAB staining.

### Mice Xenograft Model

Nude mice (28 days old) were provided by Sun Yat-sen University Laboratory Animal Center (Guangzhou, China). 5 × 10^6^ BIU-87 cells were administered into the right flanks of nude mice to establish xenograft models. The injected cells had been stably transfected with shRNA for Control and shRNA for circCA12. Tumor sizes were then determined with a Vernier caliper, and micro-metastasis nodules were counted.

### Statistical Analysis

All data are given as mean ± standard deviation (SD) and analyses were implemented in GraphPad Prism Software (V.8, La Jolla, CA, United States). One-way analysis of variance (ANOVA) along with Student’s *t*-test were implemented to compare the data. *P* < 0.05 signified statistical significance.

## Results

### Differential Expression and Circular Characterization of CircCA12 in Bladder Cancer

**FIGURE 1:**
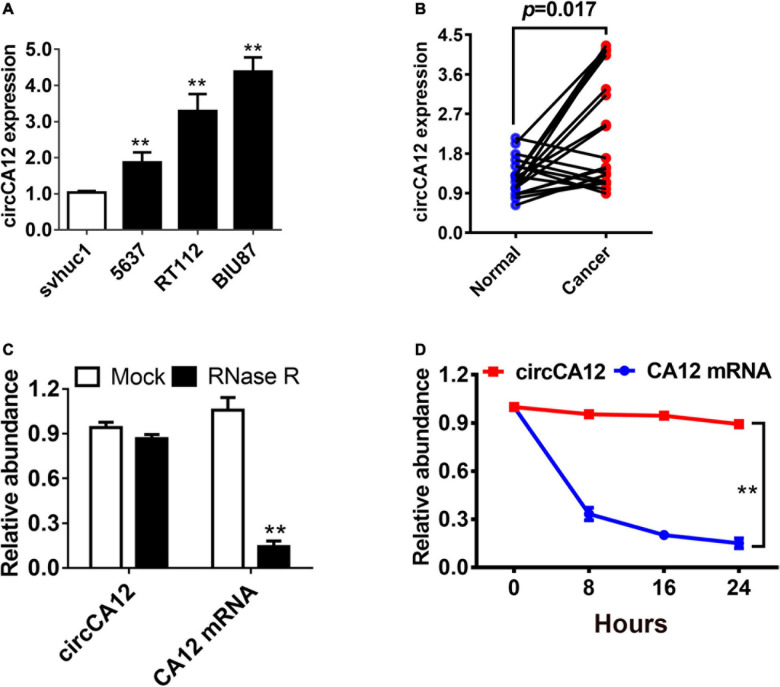
Differential expression and circular characterization of circCA12 in bladder cancer. **(A)** circCA12 relative expression in non-malignant bladder epithelial cells and bladder cancer cell lines. **(B)** circCA12 differential expression was verified in 20 bladder cancer tissues and paired non-malignant tissues. **(C)** qRT-PCR assessment of circCA12 and CA12 mRNA in BIU87 cells after RNase R treatment. **(D)** qRT-PCR assays of circCA12 and CA12 mRNA in BIU87 cells after Actinomycin D treatment. **P < 0.01.

We explored circCA12 expression level using four bladder cell lines. Results demonstrated upregulation of expression of circCA12 in UBC cell lines including 5637, RT112, and BIU-87 in contrast with svhuc1 cells (Figure 1A). We also assessed circCA12 levels in 20 pairs of UBC tissues along with their neighboring non-malignant tissue. Among them, 70% (14/20) of the samples showed increased circCA12 contents (Figure 1B; *P* = 0.0017), illustrating elevated relative expression of circCA12 in UBC samples. Resistance to the digestive effect of RNase R exonuclease confirmed the circularization of circCA12 (Figure 1C). In addition, Actinomycin D data illustrated that the half-life of circCA12 mRNA was more than 24 h, implying that the stability of the isoform is higher relative to the linear CA12 mRNA in BIU87 cells (Figure 1D).

### CircCA12 Promotes Cellular Growth and Metastasis in Bladder Cancer

**FIGURE 2:**
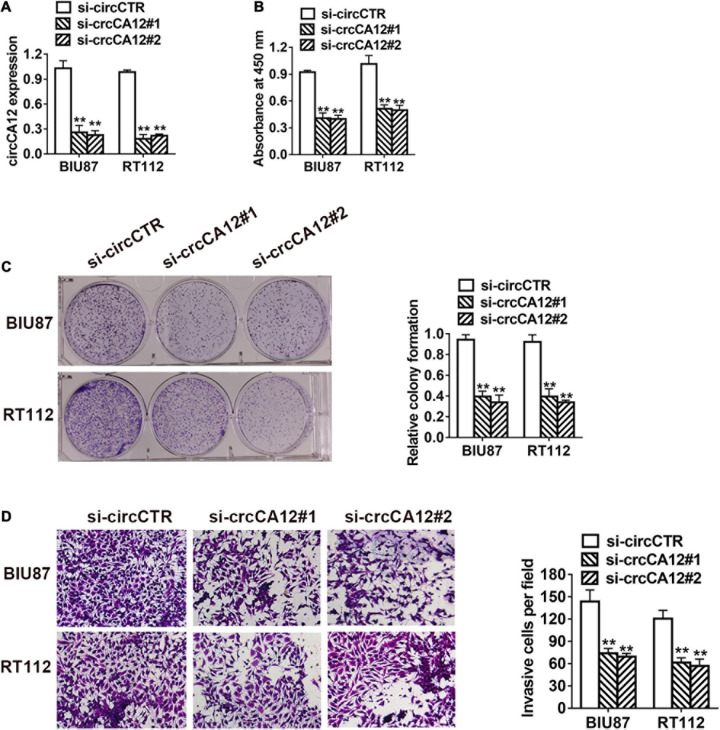
CircCA12 promotes cellular growth along with metastasis in bladder cancer. **(A)** The knockdown of circCA12 determined using qRT-PCR. **(B)** CCK-8 assay of cell proliferation. **(C)** Colony formation assays. **(D)** Suppression of circKIF4A decreased the infiltration potential in the Transwell assay. **P < 0.01.

To study the role of circCA12 in UBC, we knocked down circCA12 using si-RNA assay and verified its efficiency in two cell lines as indicated in Figure 2A. CCK-8 assay results indicated that silencing circCA12 inhibited UBC cell proliferation (RT-112 as well as BIU-87) as indicated in Figure 2B. Suppressing circCA12 significantly curtailed UBC cell colony formation as demonstrated in Figure 2C. Moreover, Transwell assay data indicated that suppression of circCA12 decreased UBC cell’s infiltration, as illustrated in Figure 2D.

### CircCA12 Knockdown Suppresses Growth and Metastasis of Bladder Cancer *in vivo*

**FIGURE 3:**
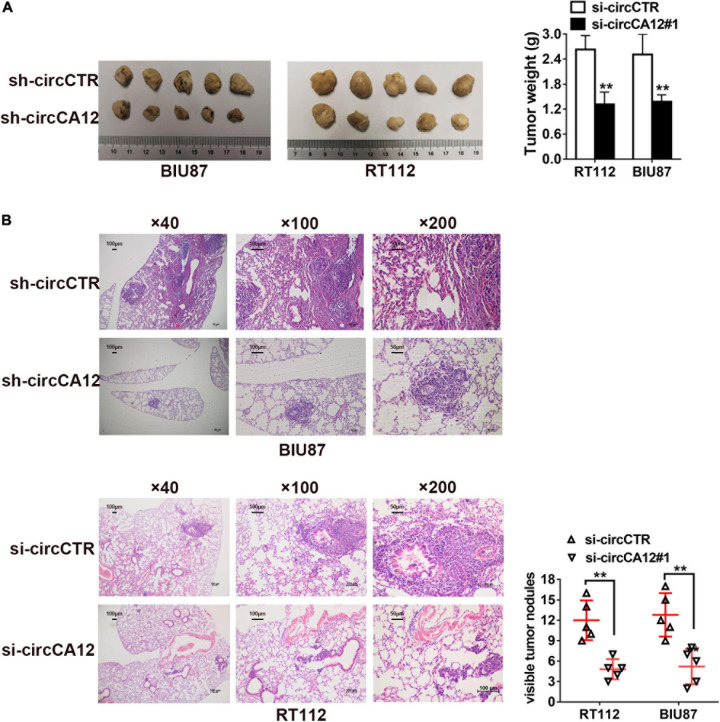
Knockdown of circCA12 represses the proliferation along with metastasis of bladder cancer *in vivo*. **(A)** Mice xenograft models were constructed using RT-112 and BIU-87 cell lines. The weight of tumors was measured after 28 days. **(B)** Lung metastases HE staining images illustrating metastatic nodule numbers in the lung. **P < 0.01.

We established mice xenograft models for further evaluation of the roles of circCA12 in UBC. Results revealed that the tumor weight of circCA12 knockdown group was remarkably reduced in contrast with the control group, as indicated in Figure 3A. Next, we prepared lung metastasis sections (4 μm) and used them to determine the impact of circCA12 on cancerous metastasis. Repression of circCA12 remarkably diminished the number of lung metastatic nodules (Figure 3B).

### CircCA12 Sponges miR-1184

**FIGURE 4:**
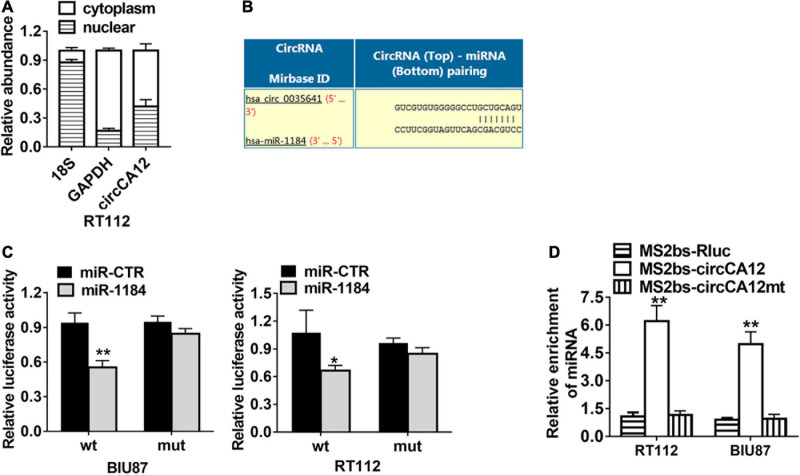
CircCA12 serves as a sponge for miR-1184. **(A)** 18S, GAPDH, and circCA12 expressions levels in the cytoplasmic and nuclear fractions. **(B)** Prediction of the docking sites of miR-1184 in the circCA12 sequence (https://circinteractome.nia.nih.gov). **(C)** Luciferase reporter assay results. RT-112 and BIU-87 cells were transfected with miR-1184 mimics, accompanied by wild-type or mutant-type circCA12 luciferase reporter assay. The predicted miR-1184 binding sites of circCA12 was mutated. **(D)** MS2-based RIP assay in RT-112 and BIU-87 cells inserted with MS2bs-circCA12, MS2bs-circCA12-mt, or MS2bs-Rluc via transfection. *P < 0.05, **P < 0.01.

We employed the Circular RNA Interactome web resource to identify possible circRNA and miRNA cross talks. From our results, we selected miR-1184 for further research (Figure 4B). CircCA12 was mainly localized in the cytoplasm (Figure 4A). Dual luciferase enzyme reporter assay demonstrated that relative luciferase enzyme activity was abated after transfection with wild-type circCA12, as well as miR-1184 mimics. The predicted miR-1184 binding sites of circCA12 was mutated (Figure 4C). Moreover, RNA immunoprecipitation (RIP) assay results revealed that miR-1184 were abundant in the non-mutant MS2bs-circCA12 group (Figure 4D).

### CircCA12 Enhances Bladder Cancer Development Through the circCA12/miR-1184/RAS Family Pathway

**FIGURE 5:**
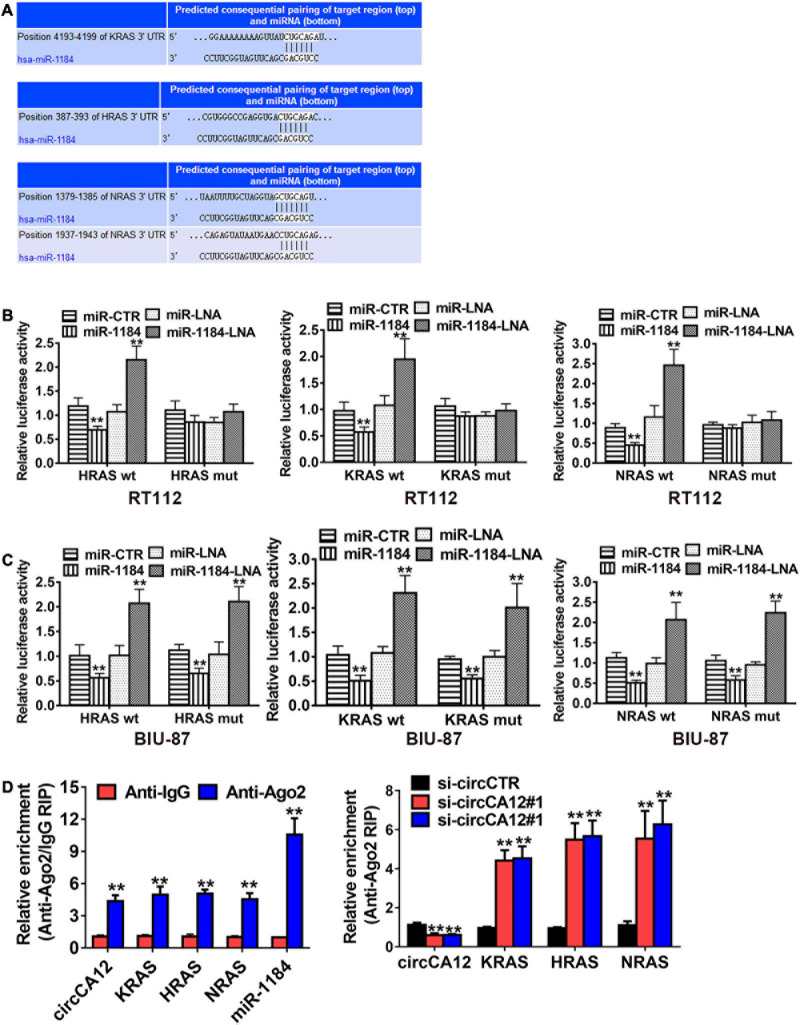
CircCA12 enhances bladder cancer development through the circCA12/mir-1184/RAS family pathway. **(A)** Docking sites of miR-1184 in the 3′-UTR of KRA/HRA/NRAS predicted by TargetScan web resource (http://www.targetscan.org). **(B,C)** Luciferase enzyme reporter analysis. We transfected miR-1184 mimics, locked nucleic acid (LNA), as well as control groups into circCA12 wild type or mutant luciferase reporter in RT-112 and BIU-87 cells. The predicted miR-1184 binding sites of 3′-UTR of HRAS/KRAS/NRAS were mutated. **(D)** RIP assay. CircCA12, miRNA-1184, and KRAS/HRAS/NRAS were abundant in the Ago2 complex in contrast with the lgG group, and enrichment of KRAS/HRAS/NRAS was elevated after silencing circCA12. CircCA12, miRNA-1184, and KRAS/HRAS/NRAS expression were measured by RT-qPCR analysis. **P < 0.01.

We then employed the TargetScan web resource^1^ to identify the down-stream miR-1184 targets. Results identified RAS family genes, including *KRAS*, *NRAS*, and *HRAS*, as the potential targets of miR-1184 (Figure 5A). Luciferase enzyme reporter assays illustrated that the relative luciferase enzyme activity was diminished after transfection with miR-1184 mimics, as well as the non-mutant RAS family (Figure 5B). On the other hand, the relative luciferase activity was increased when the miR-1184 and wild-type 3′-UTR-RAS family combination was blocked (Figure 5C). Moreover, Ago2-related RIP assays revealed that low-expression of circCA12 significantly boosted the enrichment of Ago2 by the RAS family (Figure 5D).

## Discussion

CircRNAs are comprised of recently identified non-coding RNAs that have become a hotspot in medical biotechnology. Previous investigations have documented that circRNAs’ expression is cell type-distinct, as well as tissue-distinct, and the differential expression of some circRNAs could function as potential biomarkers, thereby aiding in diagnosis and therapy of various kinds of cancer (Guo et al., 2014). Urinary bladder cancer (UBC) is the most frequently diagnosed cancer of the urinary system, as well as one of the most commonly diagnosed cancers globally (Miyazaki and Nishiyama, 2017). Several researches have documented that circRNAs can exert remarkable influence in the malignant procession of UBC. Herein, we discovered a potential circular RNA, circCA12, and determined its expression level in bladder cell lines and tissues. Our data illustrated that the relative expression of circCA12 was elevated in the cancerous part in contrast with non-malignant bladder epithelial cells and tissues, suggesting that over expression of circCA12 may be involved in the malignant cascade of UBC.

Firstly, we explored differential circCA12 levels in UBC cells and tissues. Results illustrated circCA12 upregulation in the UBC cells, as well as tissues, implying that circCA12 is potentially linked to UBC onset. Secondly, we silenced circCA12 expression in RT112, as well as BIU-87 UBC cell lines, and used them to conduct functional experiments. We found that down-regulation of circCA12 significantly repressed the growth, migration, and infiltration of UBC cells. Thereafter, animal assays were employed to further confirm our results *in vivo*. Mice xenograft models were established, followed by resection of the cancerous nodule. The weight of the tissue obtained from mice treated with silenced circCA12 expression was remarkably lower in contrast with the control group. Our *in vitro* along with the *in vivo* data demonstrated that circCA12 promotes the malignant process of bladder cancer.

Recent research evidence demonstrates that the localization of circRNAs in cytoplasm is closely associated with miRNA sponge (Hansen et al., 2013; Chen, 2016; Ye et al., 2019). The nuclear and cytoplasmic fraction assays conducted in this study verified that circACVR2A was distributed predominantly in the cytoplasm. Moreover, RIP and dual-luciferase reporter assays indicated that circCA12 could cross talk with miR-1184 in UBC cells. Similar results were obtained when circCA12 mutant-type or wild-type plasmids were transfected into UBC cells. Theoretically, miRNAs post-transcriptionally diminish the expression profiles of targeted genes by occupying the 3′UTR of the targeted mRNAs, thereby halting the translation procession or degrading the mRNA strand (Bartel, 2018; Chen et al., 2018). The TargetScan algorithm identified the RAS oncogene family as the potential downstream target of miR-1184. The human genome possesses three RAS genes (KRAS, HRAS, and NRAS), which code for the RAS proteins. RAS proteins, RAS superfamily members of small GTPases, are found at the center of a highly complex signaling network, which modulates numerous aspects of fundamental cellular processes consisting of cell survival, apoptosis, differentiation, and proliferation (Zhou et al., 2016). Moreover, RAS genes modulate human oncogenesis (Karnoub and Weinberg, 2008). In particular, functional activation of RAS genes by point mutations has been reported in approximately 30% of all human cancers (Pylayeva-Gupta et al., 2011). Recent bladder cancer studies have shown that genetic mutations in bladder epithelial cells frequently occur in *FGFR3*, *PIK3CA*, and *RAS* (all associated with *RAS* signaling networks) (Bockorny et al., 2018; Tsujino et al., 2019). In addition, a 2020 study demonstrated that the RAS mutation can be a favorable biomarker for diagnosing cancer patients (Santha et al., 2020). With regard to the relationship between RAS oncogene and microRNAs (miRNAs), accumulating evidence suggests that some miRNAs directly target *RAS* signaling pathways, thereby impeding RAS-driven tumorigenesis (Jinesh et al., 2018; Ding et al., 2020). Armed with the knowledge that circRNAs primarily serve as miRNA sponges to exert diverse biological effects, we hypothesized that the underlying mechanism through which RAS promotes UBC may be associated with ceRNA function (Thomson and Dinger, 2016). Thus, we conducted Ago2-related RIP and luciferase reporter assays in order to verify our hypothesis. Our results indicated that miR-1184 promotes tumor progression by directly targeting KRAS/NRAS/HRAS in UBC.

## Conclusion

In conclusion, results obtained herein illustrated upregulation of circCA12 in bladder cancer and was closely linked to the malignant process. MicroRNA-1184 was identified as a special target of circCA12, and circCA12 sponge acts on miRNA-1184 to abate its biological functions. We also identified the RAS oncogene family (*KRAS*, *NRAS*, and *HRAS*) as a bladder cancer stimulus factor. This study has discovered that miRNA-1184 interacts with *KRAS*, *NRAS*, and *HRAS* coding genes, thereby regulating their effect in bladder cancer.

## Data Availability Statement

The raw data supporting the conclusions of this article will be made available by the authors, without undue reservation.

## Ethics Statement

The studies involving human participants were reviewed and approved by Ethics Committee of Sun Yat-sen University Cancer Centre (SYSUCC). The patients/participants provided their written informed consent to participate in this study. The animal study was reviewed and approved by Ethics Committee of Sun Yat-sen University Cancer Centre (SYSUCC).

## Author Contributions

YL and XW designed the all experiments. LJ conducted the experiments. HT gathered specimens. XL, CH, ZL, and FZ analyzed the data. LJ, YL, and XW wrote and revised the manuscript. All authors read and approved the final manuscript.

## Conflict of Interest

The authors declare that the research was conducted in the absence of any commercial or financial relationships that could be construed as a potential conflict of interest. The reviewers HT and YZ declared a shared affiliation, with no collaboration, with several of the authors LJ, YW, HT, XL, CH, ZL, FZ, and YL, to the handling editor at the time of review.

## Footnotes

1 http://www.targetscan.org
